# Validation and optimization of classification criteria for childhood-onset systemic lupus erythematosus in a multi-center Chinese cohort

**DOI:** 10.3389/fimmu.2025.1611349

**Published:** 2025-07-17

**Authors:** Yudi Zhang, Tian Shen, Sihao Gao, Junxia Yan, Jingcheng Shi, Xuemei Tang, Mo Wang, Jun Yang, Haiguo Yu, Huawei Mao, Lanjun Shuai, Yongzhen Li, Yan Cao, Xiaoyan Li, Ying Wang, Qian Liu, Hongmei Song, Xiaochuan Wu

**Affiliations:** ^1^ Department of Pediatrics, The Second Xiangya Hospital, Central South University, Changsha, Hunan, China; ^2^ Department of Pediatrics, Peking Union Medical College Hospital, Chinese Academy of Medical Sciences, Beijing, China; ^3^ Department of Epidemiology and Health Statistics & Hunan Provincial Key Laboratory of Clinical Epidemiology, XiangYa School of Public Health, Central South University, Changsha, Hunan, China; ^4^ Department of Epidemiology and Health Statistics, Xiangya School of Public Health, Central South University, Changsha, Hunan, China; ^5^ Department of Rheumatology and Immunology, Children’s Hospital of Chongqing Medical University, Chongqing, China; ^6^ Department of Nephrology and Rheumatology, Children’s Hospital of Chongqing Medical University, Chongqing, China; ^7^ Department of Rheumatology and Immunology, Shenzhen Children’s Hospital, Shenzhen, China; ^8^ Department of Rheumatology and Immunology, Children’s Hospital of Nanjing Medical University, Nanjing, China; ^9^ Department of Immunology, Beijing Children’s Hospital, Capital Medical University, National Center for Children’s Health, Beijing, China

**Keywords:** childhood-onset systemic lupus erythematosus, classification criteria, SLICC-2012, EULAR/ACR-2019, criteria validation, criteria optimization

## Abstract

**Objective:**

This study evaluated the diagnostic accuracy of the 2012 SLICC and 2019 EULAR/ACR criteria in Chinese cSLE patients and aimed to develop an optimized classification schema based on the 2019 EULAR/ACR criteria, specifically tailored for cSLE.

**Methods:**

Data from cSLE and control cases were extracted from the CAPRID database. Gold-standard diagnosis were established by consensus among 43 rheumatologists (≥80% agreement). From 1,390 consensus cases, a random selection of 1,045 cases (512 cSLE/533 non-cSLE) were allocated to derivation (n=522) and validation (n=523) cohorts. The 2012 SLICC and 2019 EULAR/ACR criteria were evaluated in the total cohort. Multiple optimization schemes were then developed through LASSO regression with expert consultation in the derivation cohort. All potential optimization schemes underwent validation in the validation cohort, from which the optimal scheme was selected and further evaluated in an ANA-positive subgroup.

**Results:**

The 2012 SLICC criteria demonstrated sensitivity of 96.7% and specificity of 96.5%, while the 2019 EULAR/ACR criteria had sensitivity of 95.3% and specificity of 97.8%, with an optimal total score threshold of 10. When both the non-scarring alopecia and arthritis criteria were removed alongside the redefined urinary protein criterion, specificity significantly improved to 99.3% (*P* < 0.05), while sensitivity remained unaffected at 94.1% (*P* = 0.210). In the ANA-positive cohort, the optimized integrated scheme significantly improved specificity (97.7% vs. 86.4%, *P* = 0.012) while maintaining comparable sensitivity (96.2% vs. 97.8%, *P* = 0.138).

**Conclusion:**

Both criteria performed well in Chinese cSLE patients. Optimizing the 2019 EULAR/ACR criteria by removing alopecia and arthritis criteria and modifying the urinary protein criterion enhanced specificity without compromising sensitivity.

## Introduction

Systemic lupus erythematosus (SLE) is a complex autoimmune disorder characterized by immune dysregulation and chronic inflammation, with an overall mortality rate higher than that of the general population (1, 2). Childhood-onset systemic lupus erythematosus (cSLE), defined as SLE diagnosed before the age of 18, has an incidence rate of 3.97 cases per 100,000 person-years (95% CI: 3.93-4.01) in China (3). The most widely used classification criteria for both adult-onset SLE (aSLE) and cSLE are the 2012 Systemic Lupus International Collaborating Clinics (SLICC) criteria and the 2019 European League Against Rheumatism (EULAR)/American College of Rheumatology (ACR) criteria (4, 5). These criteria were primarily developed and validated in non-Asian aSLE populations. Subsequently, a dedicated study validated the applicability of the 2019 EULAR/ACR criteria in antinuclear antibody (ANA)-positive aSLE patients in China, demonstrating excellent classification performance (6). However, no specific classification criteria have been established for cSLE, as existing criteria are primarily derived from aSLE populations. Although multiple studies have evaluated the applicability of these classification criteria in cSLE, these results remain controversial (7–10).

It should be particularly noted that cSLE exhibits complex pathogenesis and demonstrates significant differences compared to aSLE, including distinct sex distribution patterns, more aggressive disease phenotypes, lower treatment response rates, and poorer prognosis (11, 12). A Turkish cohort study (13) revealed that cSLE patients have higher frequencies of renal, mucocutaneous, hematologic, and neuropsychiatric involvement, along with elevated positivity rates for anti-double-stranded DNA (anti-dsDNA) antibodies and anticardiolipin antibodies. These findings were further corroborated by a Canadian cohort study (14), which demonstrated significantly increased rates of neuropsychiatric manifestations and anticardiolipin antibody positivity in cSLE. Epidemiological data from both France and China (3, 15) consistently indicate that cSLE patients exhibit higher rates of renal and hematologic involvement, as well as greater disease severity. These differences not only underscore the importance of early diagnosis and treatment for cSLE, but also reveal the inherent limitations of applying adult-derived classification criteria to pediatric populations.

Therefore, this study aims to validate the applicability of the 2012 SLICC and 2019 EULAR/ACR classification criteria in Chinese cSLE and to explore potential optimizations to develop more appropriate classification criteria tailored to cSLE.

## Patients and methods

**Figure 1:**
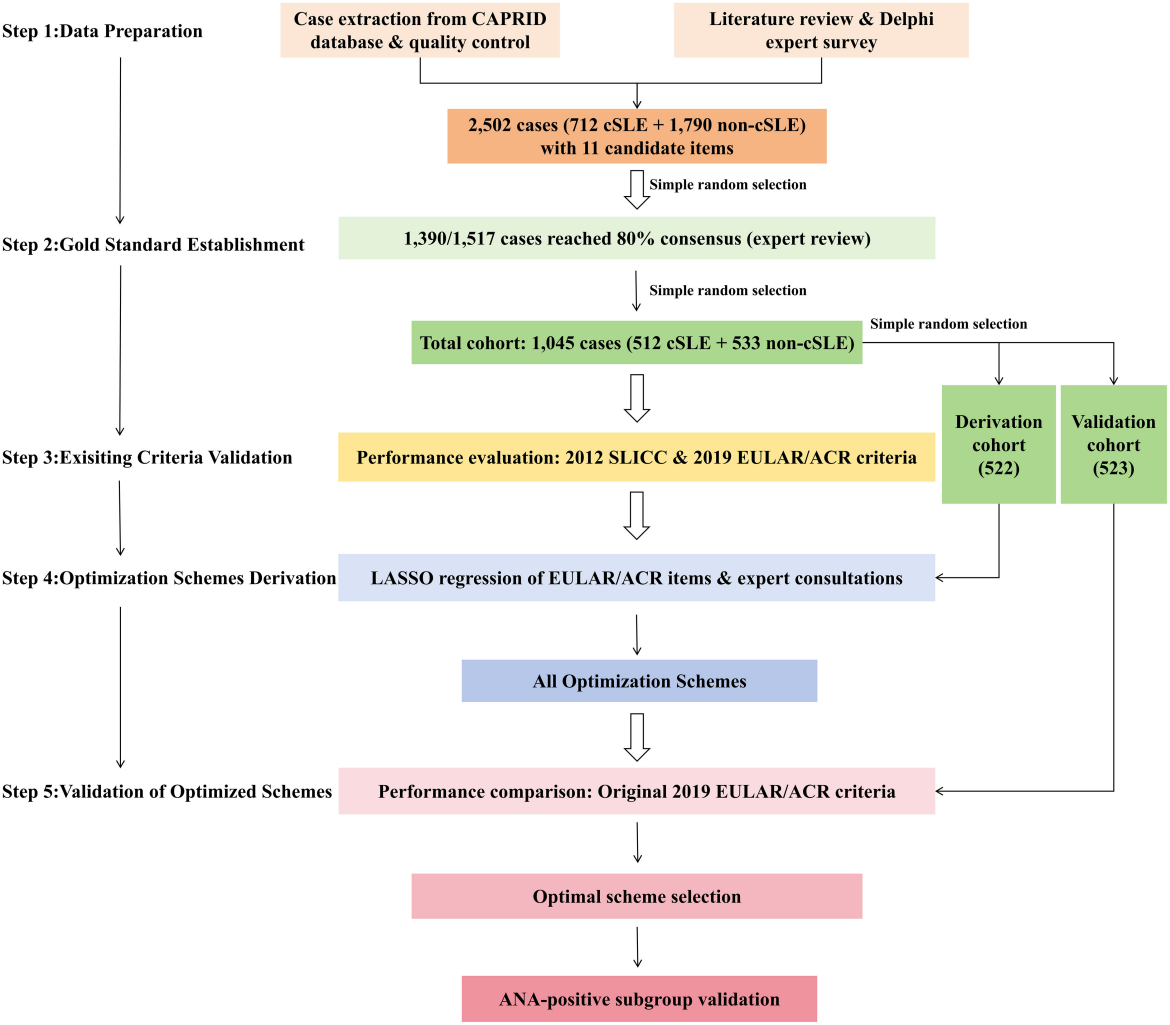
Methodological workflow for cSLE classification criteria optimization. cSLE, childhood-onset systemic lupus erythematosus; ANA, antinuclear antibody.

Using a methodological workflow grounded in clinical epidemiology and expert consensus, the optimized cSLE classification criteria were developed in five phases (**Figure 1**): (1) data preparation, (2) gold standard establishment, (3) existing criteria validation, (4) optimization schemes derivation, and (5) validation of optimized schemes.

### Stage 1: data preparation

Retrospective case data of pediatric rheumatic and immunological diseases were extracted from the Chinese Alliance of Pediatric Rheumatic and Immunologic Diseases (CAPRID) database, which comprises data from six Class-A tertiary hospitals. Cases included patients with an initial diagnosis of cSLE and those with other pediatric rheumatic diseases. Inclusion criteria were: age 5–18 years, confirmed diagnosis between 2017- 2023, and at least one year of follow-up. Exclusion criteria included neonatal lupus, monogenic lupus, overlap syndromes, infectious diseases, neoplastic diseases, and recurrent cases. After rigorous quality control procedures (including data completeness verification and outlier removal), we identified 2,502 eligible cases (712 cSLE/1,790 non-cSLE). From this pool, 1,517 cases (60.6%) were randomly selected for expert review using computer-generated randomization. Eleven supplementary candidate items were identified through systematic literature review and three-round Delphi surveys.

### Stage 2: gold standard establishment

A panel of 43 rheumatologists with over ten years of clinical experience independently evaluated de-identified cases through a standardized electronic case report form (CRF). To eliminate institutional bias, reviewers were systematically excluded from evaluating cases originating from their own hospitals. For each case, experts selected one of three predefined classifications: (1) definite cSLE, (2) non-cSLE, or (3) indeterminate. A case was considered gold-standard confirmed when ≥80% of reviewers (≥4 of 5 raters) reached concordant classification. This rigorous process yielded 1,390 diagnostically validated cases (91.6% consensus rate). From these 1,390 confirmed cases, we randomly selected 1,045 cases (512 cSLE/533 non-cSLE) to form the total study cohort. This cohort was then randomly split into a derivation cohort (n=522) and a validation cohort (n=523) for subsequent analysis.

### Stage 3: existing criteria validation

All analyses were performed using SPSS Statistics version 26.0 and R software version 4.1.1. Continuous variables were assessed for normality using Shapiro-Wilk tests. Continuous variables were assessed for normality using the Shapiro-Wilk test. Normally distributed data are presented as mean ± standard deviation (SD) and analyzed using independent t-tests (for two-group comparisons) or one-way analysis of variance (ANOVA) (for multi-group comparisons). Non-normally distributed data are expressed as median (interquartile range, IQR) and compared using Mann-Whitney U tests (two groups) or Kruskal-Wallis tests (multiple groups). Categorical variables are reported as counts (percentages) and analyzed using χ² tests or Fisher’s exact tests, as appropriate. All statistical tests were two-tailed with a significance level of α=0.05. The classification performance of the 2012 SLICC and 2019 EULAR/ACR criteria was evaluated in the total cohort. We calculated sensitivity, specificity, positive predictive value (PPV), negative predictive value (NPV), Youden index, and accuracy, with 95% confidence intervals (CIs) estimated using Wilson’s method. Receiver operating characteristic (ROC) curve analysis was conducted for the 2019 EULAR/ACR criteria to determine the optimal score threshold.

### Stage 4: optimization schemes derivation

In the derivation cohort (n = 522), we employed a dual approach combining statistical modeling and expert consensus to optimize the 2019 EULAR/ACR criteria. First, least absolute shrinkage and selection operator (LASSO) regression analysis was performed using R 4.1.1 software. The optimal penalty parameter (λ) was determined based on the minimum mean squared error (MSE) through 10-fold cross-validation, and variables with coefficients of zero were selected as optimizable items. The expert panel then discussed and identified potential optimization schemes.

### Stage 5: validation of optimized schemes

All potential optimization schemes were compared with the original 2019 EULAR/ACR criteria in the validation cohort (n=523) using sensitivity, specificity, and ROC analyses. The optimal scheme was subsequently validated in an ANA-positive subgroup.

## Results

### Demographic and clinical characteristics of included study participants

A total of 512 cSLE patients and 533 controls were included in this study. **Table 1** summarizes the disease distribution in the control group, with juvenile idiopathic arthritis (JIA) accounting for 41.3% and Henoch-Schönlein purpura (HSP) for 33.4%, representing the primary differential diagnoses. The remaining 25.3% of cases included Kawasaki disease, juvenile dermatomyositis, ANCA-associated vasculitis, necrotizing lymphadenitis, Takayasu’s arteritis, rheumatic fever, and other miscellaneous disorders.

**Table 1:** Diseases constitution of the control group.

Diseases, n (%)	Cases (n=533)
Juvenile idiopathic arthritis	220 (41.3)
Henoch-Schonlein purpura	178 (33.4)
Kawasaki disease	36 (6.8)
Juvenile dermatomyositis	36 (6.8)
ANCA-associated vasculitis	11 (2.1)
Necrotizing lymphadenitis	9 (1.7)
Takayasu’s arteritis	8 (1.5)
Rheumatic fever	7 (1.3)
Sjogren’s syndrome	5 (0.9)
Scleroderma	5 (0.9)
Behcet’s disease	4 (0.8)
Primary antiphospholipid syndrome	3 (0.6)
Others	11 (2.1)

Demographic characteristics of the total cohort are presented in **Table 2**. The cSLE group was older (11.0 (3.0) *vs* 9.0 (6.0), *P* < 0.001) and had higher female predominance (82.8% *vs* 43.0%, *P* < 0.001) compared to controls. Additionally, disease duration at diagnosis was slightly longer in cSLE (1.00 *vs* 0.90, *P* = 0.004).

**Table 2:** Demographic and clinical characteristics of the total cohort.

Characteristics	cSLE group (n=512)	Control group (n=533)	*P*-value
Age, years (IQR)	11.0 (3.0)	9.0 (6.0)	<0.001
Gender, n (%)			<0.001
Female	424 (82.8)	229 (43.0)	
Male	88 (17.2)	304 (57.0)	
Course of disease, months (IQR)	1.00 (1.50)	0.90 (1.64)	0.004

IQR, Interquartile range.

**Table 3** compares clinical and immunological characteristics between groups. All cSLE classification criteria manifestations (e.g., fever, neuropsychiatric, joint involvement, renal involvement) and immunological characteristics (e.g., ANA, anti-dsDNA antibodies, complement levels) were significantly more frequent in cSLE (all *P* < 0.001). Among candidate items, cSLE showed higher positivity rates of autoimmune family history, Raynaud’s phenomenon, interstitial lung disease, and autoimmune hepatitis (*P* < 0.05), but no differences in diffuse alveolar hemorrhage, autoimmune hyperthyroidism, autoimmune pancreatitis, or mesenteric vasculitis (*P* > 0.05).

**Table 3:** Clinical and immunological characteristics of cSLE group and control group.

Characteristics, n (%)	cSLE group (n=512)	Control group (n=533)	*X^2^*	*P*-value
Clinical items*				
Fever	193 (37.7)	88 (16.5)	180.164	<0.001
Leukopenia	238 (46.5)	18 (3.4)	262.360	<0.001
Thrombocytopenia	140 (27.3)	10 (1.9)	137.776	<0.001
Autoimmune hemolysis	229 (44.7)	2 (0.4)	295.755	<0.001
Neuropsychiatric	40 (7.8)	5 (0.9)	29.948	<0.001
Non-scarring alopecia	100 (19.5)	6 (1.1)	97.060	<0.001
Oral ulcers	118 (23.1)	26 (4.9)	72.557	<0.001
Cutaneous lupus	150 (29.3)	16 (3.0)	135.133	<0.001
Serosal	213 (41.6)	45 (8.4)	154.425	<0.001
Joint involvement	112 (21.9)	226 (42.4)	50.281	<0.001
Renal involvement	349 (68.2)	111 (20.8)	237.481	<0.001
Immunology items*				
Antinuclear antibodies	499 (97.5)	88 (16.5)	698.105	<0.001
Antiphospholipid antibodies	139 (27.2)	15 (2.8)	123.069	<0.001
Complement proteins	485 (94.7)	81 (15.2)	665.301	<0.001
Anti-dsDNA antibody	442 (86.3)	2 (0.4)	789.545	<0.001
Anti-Smith antibody	182 (35.6)	8 (1.5)	203.481	<0.001
Candidate items				
Autoimmune family history	32 (6.2)	18 (3.4)	4.731	0.030
Raynaud’s phenomenon	14 (2.7)	2 (0.4)	8.139	0.004
Interstitial Lung disease	23 (4.5)	5 (0.9)	11.324	<0.001
Diffuse alveolar hemorrhage	4 (0.8)	2 (0.4)	0.211	0.646
Autoimmune hepatitis	13 (2.5)	0 (0.0)	–	<0.001
Autoimmune thyroid hyperthyroidism	2 (0.4)	2 (0.4)	0.000	1.000
Autoimmune pancreatitis	4 (0.8)	0 (0.0)	–	0.057
Mesenteric vasculitis	2 (0.4)	3 (0.6)	0.000	1.000

*The definitions of Clinical and Immunology items are based on the 2019 EULAR/ACR classification criteria.

### Validation of existing classification criteria

**Figure 2:**
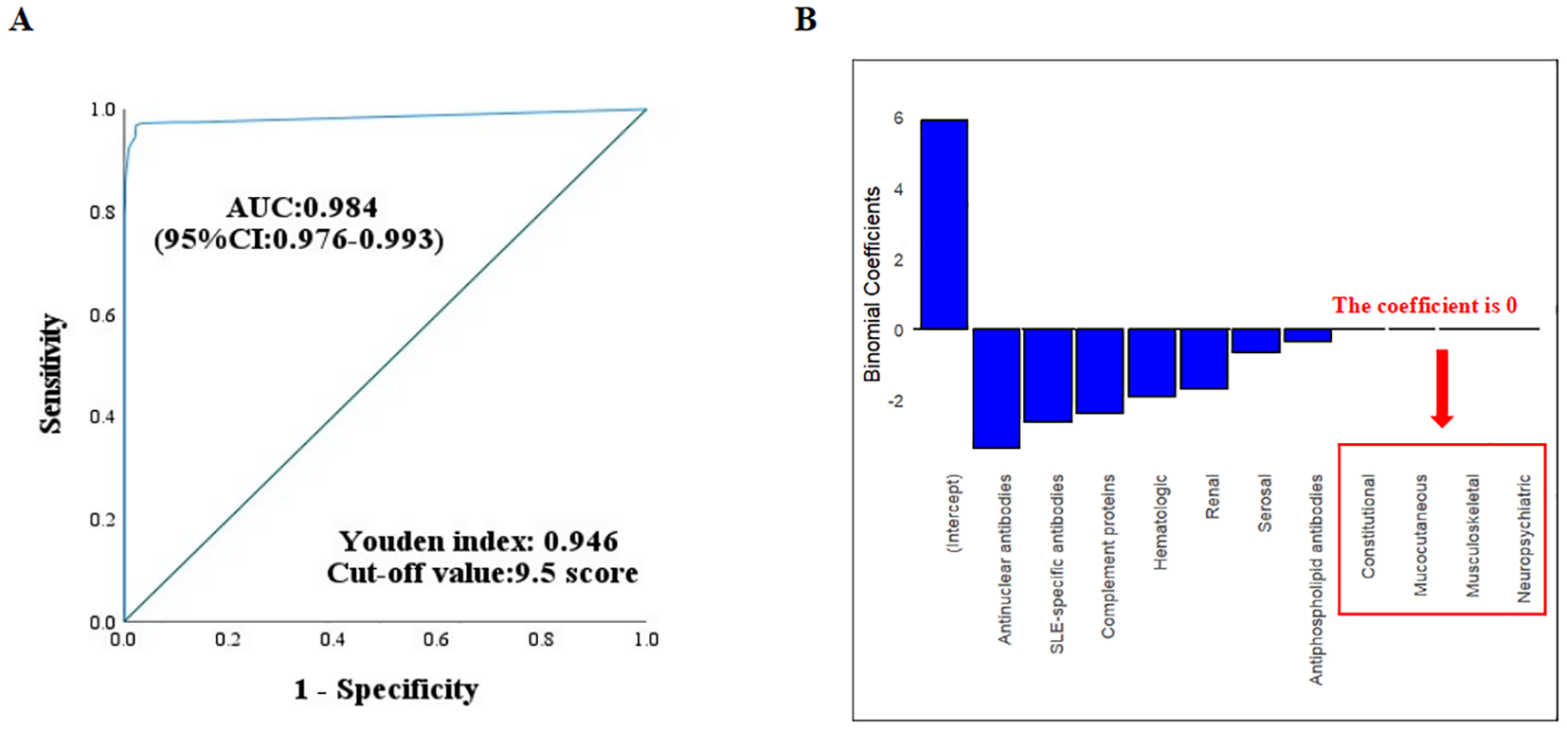
**(A)** ROC curve analysis of the 2019 EULAR/ACR criteria in the derivation cohort. The optimal cutoff value was determined by maximizing Youden’s index. **(B)** Regression coefficients for the 10 systemic domains in the 2019 EULAR/ACR criteria. Variables with 0 coefficients showed negligible weighting in the classification model.

**Table 4** evaluated the classification performance of the 2012 SLICC and 2019 EULAR/ACR criteria in the total cohort. The 2012 SLICC criteria showed 96.7% (95% CI: 94.6%-98.0%) sensitivity and 96.4% (95% CI: 94.4%-97.8%) specificity, while the 2019 EULAR/ACR criteria demonstrated 95.3% (95% CI: 93.0%-96.9%) sensitivity and 97.8% (95% CI: 96.0%-98.8%) specificity. Both criteria achieved 96.6% accuracy (Youden index=0.93). **Figure 2A** shows the ROC curve for the 2019 EULAR/ACR criteria score. The graph illustrates excellent diagnostic performance (AUC= 0.984, 95% CI 0.976-0.993), with a maximum Youden index of 0.946 corresponding to an optimal cutoff score of 10 (rounded from 9.5) for cSLE classification.

**Table 4:** Performance measures for the 2012 SLICC and 2019 EULAR/ACR criteria in the total cohort.

Criteria	Sensitivity (95% CI)	Specificity (95% CI)	PPV	NPV	Accuracy	Youden index
2012 SLICC	96.7% (94.6%-98.0%)	96.4% (94.4%-97.8%)	97.6%	95.6%	96.6%	0.93
2019 EULAR/ACR	95.3% (93.0%-96.9%)	97.8% (96.0%-98.8%)	96.3%	96.8%	96.6%	0.93
*X^2^*	1.245	1.628	–	–	–	–
*P*-value	0.265	0.202	–	–	–	–

SLICC, Systemic Lupus International Collaborating Clinics; EULAR/ACR, European League Against Rheumatism/American College of Rheumatology; CI, Confidence Interval; PPV, Positive predictive value; NPV, Negative predictive value.

### Exploration of optimization in the derivation cohort using lasso regression and expert opinions

LASSO regression analysis of the ten 2019 EULAR/ACR-defined systemic domains in the derivation cohort showed zero coefficients for constitutional, mucocutaneous, neuropsychiatric, and musculoskeletal systems (**Figure 2B**). An expert panel reviewed the findings and reached a consensus. For non-scarring alopecia, musculoskeletal system involvement, and the assessment of urinary protein results, eliminations or definitional amendments were proposed individually or in combination, resulting in various optimization strategies. Among these, the criteria for urinary protein results were revised to include one of the following: 24-hour urinary protein > 150 mg, UPr/Cr > 0.2, or a positive qualitative urinary protein test. ROC analysis determined that the optimal total score cutoff for these optimization strategies was 9.5 points.

### Evaluation of all optimization schemes in the validation cohort

In the validation cohort (n=523), all optimization schemes were systematically evaluated (**Table 5**). Individual exclusion of alopecia or arthritis criteria showed comparable sensitivities(95.3- 95.7% *vs* 96.5% original, *P* > 0.05) with modest specificity improvements (97.0-98.9% *vs* 95.9% original, *P* >0.05).The redefined proteinuria criteria maintained identical sensitivity (96.5%) and specificity (95.9%) compared to the original criteria (*P* = 1.000). However, combining the exclusion of both alopecia and arthritis criteria with the redefined proteinuria criteria significantly improved specificity to 99.3% (*P* < 0.05), while sensitivity remained comparable at 94.1% (*P* = 0.210).

**Table 5:** Comparison of classification performance between optimized schemes and the 2019 EULAR/ACR criteria in the validation cohort.

Schemes	Sensitivity (95%CI)	*X^2^*	*P*-value	Specificity (95%CI)	*X^2^*	*P*-value
2019 EULAR/ACR	96.5% (93.2%-98.3%)	–	–	95.9% (92.5%-97.8%)	–	–
Alopecia Removed	95.7% (92.2%-97.7%)	0.208	0.648	97.0% (94.0%-98.6%)	0.491	0.483
Arthritis Removed	95.3% (91.7%-97.4%)	0.447	0.504	98.9% (96.5%-99.7%)	3.594	0.058
Optimized Urine Protein	96.5% (93.2%-98.3%)	0.000	1.000	95.9% (92.5%-97.8%)	0.000	1.000
Alopecia and arthritis removed + Optimized Urine Protein	94.1% (90.3%-96.6%)	1.574	0.210	99.3% (97.0%-99.9%)	5.046	0.025

EULAR/ACR, European League Against Rheumatism/American College of Rheumatology.

### Validation of the optimized integrated scheme in ANA-positive subgroup

To further evaluate the classification accuracy, we assessed the classification performance of the optimized integrated scheme in the ANA-positive subgroup, comprising 499 cSLE cases and 88 controls. The most frequent diagnoses among controls were JIA (48.9%) and juvenile dermatomyositis (21.6%). Comparative analysis showed that the optimized integrated scheme significantly improved specificity versus the 2019 EULAR/ACR criteria (97.7% *vs* 86.4%, *P* = 0.012) while maintaining comparable sensitivity (96.2% *vs* 97.8%, *P* = 0.138) (**Table 6**).

**Table 6:** Comparison of classification performance between the optimized integrated scheme and the 2019 EULAR/ACR Criteria in the ANA-positive cohort.

Schemes	Sensitivity (95%CI)	*X^2^*	*P*-value	Specificity (95%CI)	*X^2^*	*P*-value
2019 EULAR/ACR	97.8% (96.0%-98.8%)	2.199	0.138	86.4% (77.0%-92.5%)	6.286	0.012
Optimized integrated scheme*	96.2% (94.0%-97.6%)			97.7% (91.3%-99.6%)		

*Optimized integrated scheme: removing alopecia and arthritis while redefining urinary protein quantitation.

## Discussion

This multicenter, large-sample retrospective study systematically evaluated the classification performance of the 2012 SLICC and 2019 EULAR/ACR criteria in a Chinese cSLE population. Through the integration of rigorous statistical analysis with expert consensus, we developed a revised classification scheme optimized for Chinese cSLE patients. The revised scheme significantly improved specificity while maintaining comparable sensitivity, with consistent performance in the ANA-positive subgroup. This provides clinically relevant improvements for cSLE classification in the Chinese population.

In the total cohort of this study, both the 2012 SLICC and 2019 EULAR/ACR criteria demonstrated comparable sensitivity (96.7% vs. 95.3%) and specificity (96.4% vs. 97.8%) at the time of diagnosis. Consistent with our findings, Ma et al. (16) also observed similar sensitivity and specificity between the two criteria in a retrospective cohort study of 156 cSLE cases. However, discrepancies exist among different studies. In a longitudinal study by Aslan et al. (17) involving 111 cSLE and 104 controls, the performance of three classification criteria was evaluated at diagnosis, 1-year follow-up, and final follow-up. The results showed that the 2019 EULAR/ACR criteria exhibited higher sensitivity at diagnosis and 1-year follow-up, and while its specificity was lower than that of the 1997 ACR criteria, it was superior to the 2012 SLICC criteria. A similar trend was observed in an Israeli multicenter pediatric cohort study (10), where the 2019 EULAR/ACR criteria demonstrated the highest sensitivity throughout the follow-up period, along with slightly better specificity than the 2012 SLICC criteria. However, an Omani multicenter study (9) found that although the 2019 EULAR/ACR criteria showed superior sensitivity at diagnosis, 1-year follow-up, and final follow-up, its specificity remained the lowest. Adjusting the threshold to 13 points improved specificity but at the cost of significantly reduced sensitivity.

Conversely, other studies indicated that the classification performance of the 2019 EULAR/ACR criteria was not superior to that of the 2012 SLICC criteria. A Turkish multicenter retrospective study (18) revealed that the 2012 SLICC criteria outperformed the 2019 EULAR/ACR criteria in both sensitivity and specificity. Additionally, a UK cohort study (19) demonstrated that the 2012 SLICC criteria had higher sensitivity than the 2019 EULAR/ACR criteria at both initial diagnosis and final follow-up, albeit with lower specificity. Similarly, a Brazilian single-center study (20) found that compared to the EULAR/ACR criteria (≥10 points), the SLICC criteria exhibited better specificity at initial diagnosis and 1-year follow-up, despite comparable sensitivity between the two. Raising the threshold to 13 points improved the specificity of the 2019 EULAR/ACR criteria but also led to a decline in sensitivity. While most existing studies have optimized classification criteria by adjusting thresholds, our cohort achieved ideal sensitivity and specificity at the 10-point threshold. Therefore, we focused on simplifying the classification criteria rather than modifying the score threshold, making this the first study to propose a simplified classification approach.

Despite good classification performance, the 2019 EULAR/ACR criteria have notable limitations, particularly in specificity. Requiring ANA positivity as an entry criterion limits its applicability to ANA-positive cases only, and specificity within this subgroup still needs improvement. Second, several methodological challenges emerged during the expert blind evaluation, including face-to-face discussions guided by extensive clinical experience and CRF data entry. Furthermore, direct application of criteria developed from adult populations to pediatric cohorts presents significant practical limitations. Key limitations include: (1) The rarity of typical non-scarring alopecia in pediatric populations, resulting in the absence of objective, standardized assessment criteria for alopecia in cSLE. Frequently, reported “alopecia” in medical records reflects patient or parental descriptions of “increased hair shedding” or “new-onset hair loss”, which often lack objective clinical confirmation as either alopecia areata or measurable hair density reduction upon physical examination. This introduces substantial subjectivity in assessing and documenting disease-related alopecia. Consequently, the expert panel reached consensus to consider excluding non-scarring alopecia from the optimized classification criteria.

Both the 2012 SLICC and 2019 EULAR/ACR classification criteria adopt consistent definitions for musculoskeletal involvement, mandating either: (1) synovitis involving ≥2 joints characterized by swelling or effusion; or (2) tenderness in ≥2 joints accompanied by morning stiffness persisting ≥30 minutes. Despite multiple studies (21–23) identifying musculoskeletal involvement as a common clinical feature in cSLE, only 21.9% of Chinese cSLE patients met the strict diagnostic criteria for joint involvement. Among cSLE patients classified as negative for joint involvement, common presentations included: (1) monoarticular involvement; (2) persistent arthralgia without objective swelling or radiographic evidence of effusion/synovitis; or (3) tenderness with limited range of motion but lacking the required morning stiffness duration. These clinical presentations may lead to false-negative classification of joint involvement in cSLE. To minimize selection bias, our study employed a control group representing the actual spectrum of pediatric rheumatic diseases treated across the six participating centers, rather than specifying particular disease controls. Notably, JIA was the predominant diagnosis in both the overall control group and ANA-positive subset, with 42.4% demonstrating musculoskeletal involvement-the sole criterion showing statistically significant intergroup differences, albeit lower in the SLE group. Thus, despite being a significant clinical manifestation in cSLE, musculoskeletal involvement showed zero contribution to disease classification in LASSO regression analysis. Consequently, musculoskeletal involvement was identified as a potential candidate for criteria optimization. Unsurprisingly, the proposal to exclude the arthritis criterion generated substantial discussion and controversy among the expert panel. Final analysis revealed that the optimized scheme, excluding joint involvement, maintained sensitivity while significantly improving specificity. However, we recognize that this approach may compromise sensitivity, particularly for early SLE identification.

The 2019 EULAR/ACR classification criteria define proteinuria as >500 mg/24-hour urine protein or an equivalent UPr/Cr ratio. However, pediatric rheumatology experts agree that this threshold is suboptimal for pediatric populations due to growth-related variations. Additionally, there are no established conversion factors between 24-hour urine protein and UPr/Cr for diverse pediatric ethnic groups. The optimal definition of proteinuria in children should incorporate body size adjustments, such as 24-hour urine protein/Kg or/m^2^, to enable better comparison across age groups in cSLE (24, 25). While UPr/Cr is a convenient alternative (26), complete 24-hour urine collections or UPr/Cr measurements are often unavailable in clinical practice, especially in control groups (e.g., JIA, JDM, KD) where renal involvement is uncommon. Given these challenges, we proposed a revised proteinuria definition incorporating: (1) >150 mg/24-hour urine protein; (2) UPr/Cr >0.2; or (3) positive qualitative proteinuria on routine urinalysis. This revised definition demonstrated comparable sensitivity and specificity to the original criteria. This finding aligns with the well-documented high prevalence of renal involvement and more severe organ damage in cSLE (27). We emphasize that these three methods are not fully interchangeable, and their appropriate use requires clinical judgment. The expert panel strongly affirms the clinical utility of both 24-hour urine protein quantification and UPr/Cr for evaluating renal involvement in cSLE. Future studies should establish validated, body size-adjusted cut-off values for 24-hour proteinuria and UPr/Cr specific to Chinese cSLE populations.

Despite LASSO regression analysis showing zero contribution from neuropsychiatric involvement to the classification criteria, pediatric rheumatologists argued that the reported 4.81% prevalence in Chinese cSLE patients substantially underestimates the true incidence. Globally, neuropsychiatric manifestations are reported in 13.5%-50.9% of cSLE cases (28). The reliability of children’s descriptions of neurological symptoms, such as headaches, personality changes, and memory impairment, varies significantly with age. Moreover, pediatric patients typically exhibit lower tolerance for invasive procedures like lumbar punctures compared to adults, which may contribute to underreporting of neuropsychiatric involvement. Data from a specialized center that routinely performs cerebrospinal fluid (CSF) analysis, electroencephalography (EEG), and brain positron emission tomography-computed tomography (PET-CT) in cSLE patients show frequent observation of elevated CSF pressure, non-epileptiform EEG abnormalities, and cerebral metabolic changes, even in asymptomatic patients. Notably, excluding neuropsychiatric criteria from our cohort did not improve specificity or sensitivity. Therefore, the expert panel decided to retain neuropsychiatric involvement in the refined classification criteria, based on this retrospective cohort study.

Furthermore, our analysis demonstrates that for ANA-positive cSLE patients, the most discriminative classification criteria include immunological markers and objectively quantifiable manifestations in the hematological system, kidneys, and serosal membranes. It is crucial to emphasize that classification criteria serve primarily to differentiate SLE from its mimickers, rather than comprehensively characterize the disease. Thus, our proposed optimization-removing non-scarring alopecia and arthritis while revising the proteinuria criterion-reinforces the essential classification framework for this population. Although derived from Chinese cSLE data, the optimized framework’s reliance on objective parameters (e.g., renal/hematologic metrics) and exclusion of subjective features (e.g., alopecia) may have transnational relevance. Given the biological consistency of pediatric SLE manifestations, this approach could inform global cSLE classification, though validation across ethnic populations remains imperative.

To ensure scientific rigor and objectivity, we implemented multiple measures, including data anonymization and independent expert blind review. Laboratory results were systematically extracted from a dedicated disease database using standardized codes to minimize data entry errors during CRF data entry. However, several inherent limitations remain. The primary limitation is the retrospective design, which relies on pre-existing medical records. Data completeness and accuracy are limited by the quality of source documentation, potentially affecting the precision of our results. Therefore, prospective studies are needed to validate these findings. Additionally, the small sample size of ANA-negative cSLE patients (n=14) limited our ability to conduct a detailed analysis of classification improvement in this subgroup. The proposed optimized scheme is specifically tailored for ANA-positive cSLE patients.

## Conclusion

In this large Chinese cSLE cohort, both 2012 SLICC and 2019 EULAR/ACR classification criteria demonstrated robust performance. The 2019 EULAR/ACR criteria, with a total score threshold of 10, proved equally effective for cSLE classification. Furthermore, the removal of non-scarring alopecia and arthritis, along with the modification of the urinary protein criterion based on the 2019 EULAR/ACR classification criteria, significantly improved specificity without compromising sensitivity.

## Data Availability

The raw data supporting the conclusions of this article will be made available by the authors, without undue reservation.

## Glossary

ACR: American College of Rheumatology
ANA: Antinuclear antibody
anti-dsDNA: Anti-double-stranded DNA
ANOVA: One-way analysis of variance
aSLE: Adult-onset systemic lupus erythematosus
cSLE: Childhood-onset systemic lupus erythematosus
CIs: Confidence intervals
CRF: Case report form
CSF: Cerebrospinal fluid
EEG: Electroencephalography
EULAR: European League Against Rheumatism
HSP: Henoch-Schönlein purpura
IQR: Interquartile range
JIA: Juvenile idiopathic arthritis
LASSO: Least absolute shrinkage and selection operator
MSE: Minimum mean squared error
NPV: Negative predictive value
PET-CT: Positron emission tomography-computed tomography
PPV: Positive predictive value
ROC: Receiver operating characteristic
SD: Standard deviation
SLE: Systemic lupus erythematosus
SLICC: Systemic Lupus International Collaborating Clinics.
